# Supplementary material for: Chromatin determinants impart camptothecin sensitivity
Source: EMBO Rep. 2017 Apr 7;18(6):1000–12. doi: 10.15252/embr.201643560 (PMC5452016; doi:10.15252/embr.201643560)
Supplement: Supplementary file 1 — Expanded View Figures PDF [file EMBR-18-1000-s001.pdf]

Expanded View Figures

Figure EV1. A synthetic viability screening identifies the SIR complex as the major driver of *tof1*Δ camptothecin hypersensitivity.

A Spontaneous suppressors of camptothecin sensitivity of *tof1*Δ cells.
B Suppressor strains recovered from the *tof1*Δ synthetic viability screen are G418 resistant, suggesting presence of the *TOF1* deletion cassette.
C Deletion of *IRC15* does not suppress *tof1*Δ camptothecin hypersensitivity.
D Deletion of *IME2* does not suppress *tof1*Δ camptothecin hypersensitivity.
E Deletion of *SIR2* and deletion of *TOP1* lead to similar levels of camptothecin resistance, and combining the two mutations does not further increase camptothecin resistance.
F Deletion of *HML* does not alter the camptothecin hypersensitivity of *tof1*Δ cells.
G *tof1*Δ cells and congenic wild-type cells were pre-grown either in the absence or in the presence of sirtinol. They were subsequently synchronised in G1 and released into S phase in the presence of camptothecin, either with or without sirtinol. Cell cycle progression was monitored by FACS analysis.

 © 2017 The Authors

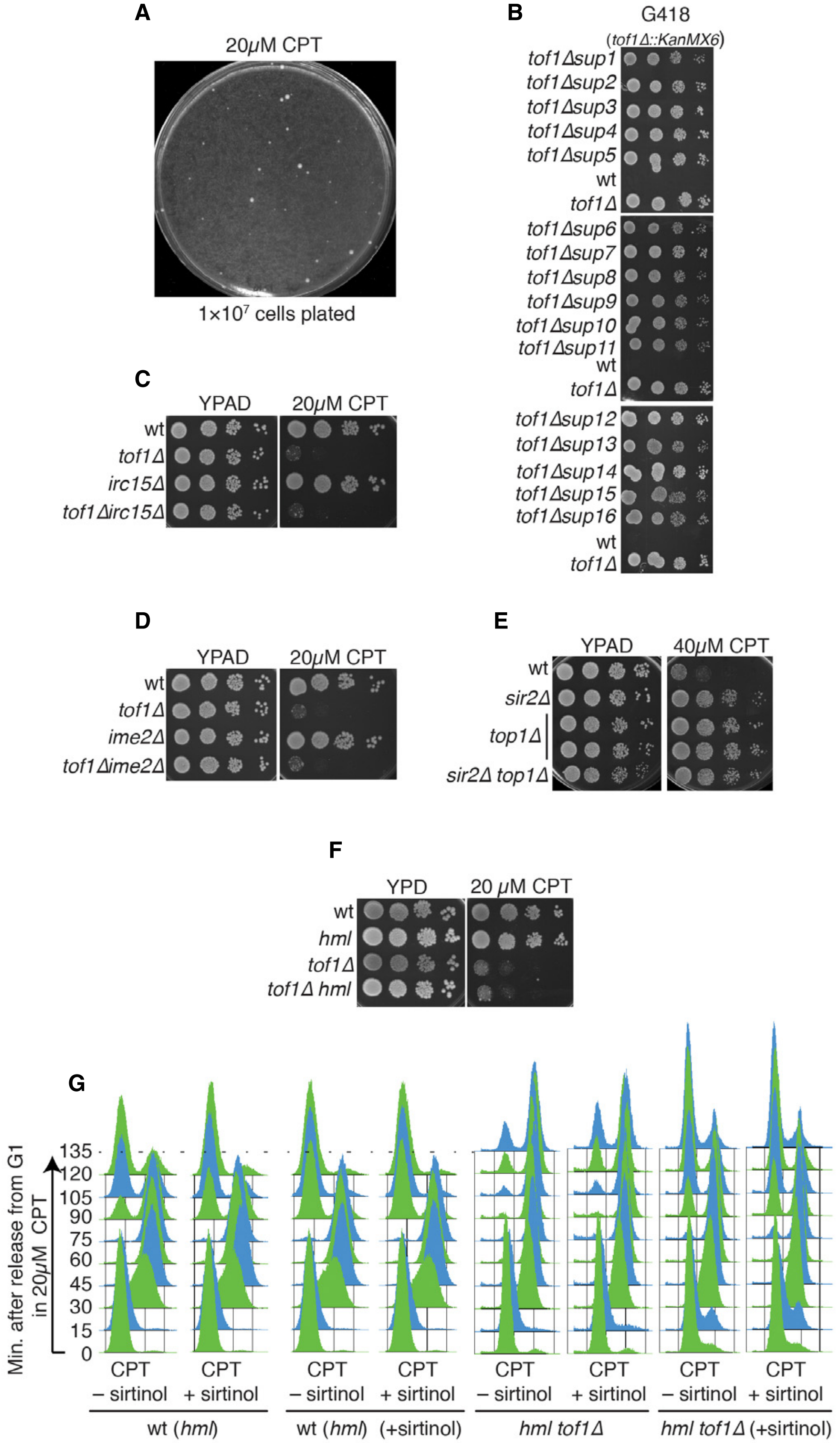
A
20μM CPT
1×10^7 cells plated
B
G418
(tof1Δ::KanMX6)
tof1Δsup1
tof1Δsup2
tof1Δsup3
tof1Δsup4
tof1Δsup5
wt
tof1Δ
tof1Δsup6
tof1Δsup7
tof1Δsup8
tof1Δsup9
tof1Δsup10
tof1Δsup11
wt
tof1Δ
tof1Δsup12
tof1Δsup13
tof1Δsup14
tof1Δsup15
tof1Δsup16
wt
tof1Δ
C
YPAD
20μM CPT
wt
tof1Δ
irc15Δ
tof1Δirc15Δ
D
YPAD
20μM CPT
wt
tof1Δ
ime2Δ
tof1Δime2Δ
E
YPAD
40μM CPT
wt
sir2Δ
top1Δ
sir2Δ top1Δ
F
YPD
20 μM CPT
wt
hml
tof1Δ
tof1Δ hml
G
Min. after release from G1 in 20μM CPT
135
120
105
90
75
60
45
30
15
0
CPT – sirtinol
CPT + sirtinol
CPT – sirtinol
CPT + sirtinol
CPT – sirtinol
CPT + sirtinol
CPT – sirtinol
CPT + sirtinol
wt (hml)
wt (hml) (+sirtinol)
hml tof1Δ
hml tof1Δ (+sirtinol)

Figure EV1.

Figure EV2. Binding of the SIR complex to highly expressed genomic loci mediates camptothecin sensitivity.

A Deletion of *SIR1* does not rescue camptothecin hypersensitivity in *rad51*Δ cells.

B Deletion of *SIR1*, *SIR2*, *SIR3 or SIR4* increases camptothecin resistance to a similar extent, both in a wild-type and in a *tof1*Δ background.

C Analysis of ChIP-seq data for the indicated proteins: enrichment is plotted as a function of the genomic coordinate; in green is the protein/modification tested; in grey are controls. The position of each ORF is indicated at the bottom of the panel and by two vertical red lines. The horizontal red lines indicate the thresholds used in this work to determine enrichment (Sir2/Sir3/Sir4/GFP/H3) or loss (H4-K16) of ChIP signal.

D The majority of SIR-positive ORFs are highly expressed genes, but high expression does not necessarily correlate with high SIR score. Vertical lines identify quartiles (I, II, III, IV) of the gene expression distribution.

 © 2017 The Authors

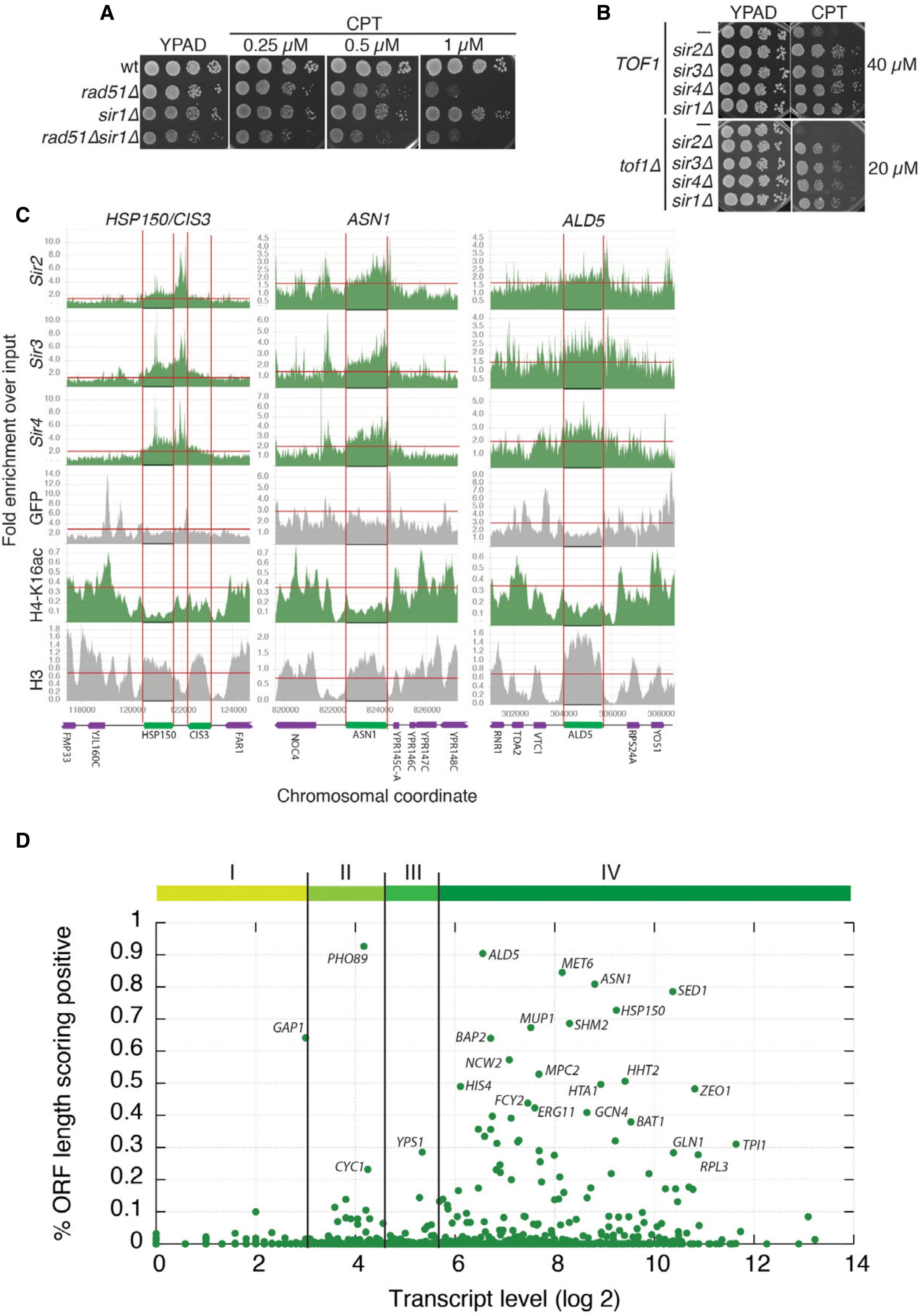
A
CPT
YPAD
0.25 μM
0.5 μM
1 μM
wt
rad51Δ
sir1Δ
rad51Δsir1Δ
B
YPAD
CPT
TOF1
sir2Δ
sir3Δ
sir4Δ
sir1Δ
40 μM
tof1Δ
20 μM
C
HSP150/CIS3
ASN1
ALD5
Sir2
Sir3
Sir4
GFP
H4-K16ac
H3
Fold enrichment over input
Chromosomal coordinate
D
I
II
III
IV
% ORF length scoring positive
Transcript level (log 2)
PHO89
GAP1
CYC1
YPS1
ALD5
MET6
ASN1
SED1
HSP150
MUP1
SHM2
BAP2
NCW2
MPC2
HHT2
HIS4
HTA1
ZEO1
FCY2
ERG11
GCN4
BAT1
GLN1
TPI1
RPL3

Figure EV2.
