# Supplementary figures and images for: Chromatin determinants impart camptothecin sensitivity
Source: EMBO Rep. 2017 Apr 7;18(6):1000–12. doi: 10.15252/embr.201643560 (PMC5452016; doi:10.15252/embr.201643560)

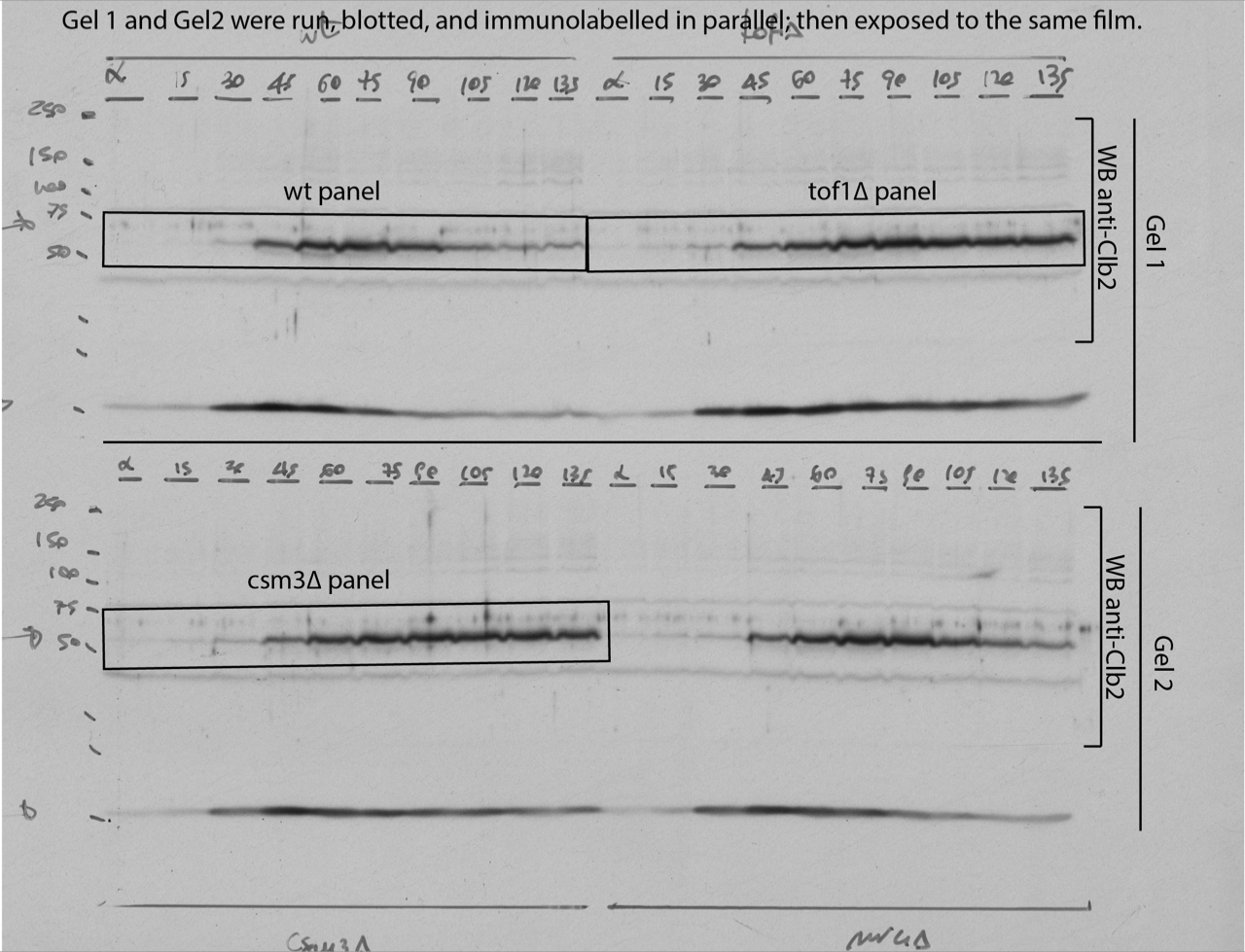

Supplement: Supplementary file 7 — Source Data for Figure 3 [file EMBR-18-1000-s006.tif]
